# 1215. The Impact of MRSA Nasal PCR on Antibiotic De-escalation

**DOI:** 10.1093/ofid/ofad500.1055

**Published:** 2023-11-27

**Authors:** Adam Silverman, Pranisha Gautam-Goyal, Patricia Saunders-Hao, Myriam Kline, Sumeet Jain

**Affiliations:** Northwell Health, Manhasset, New York; Zucker School of Medicine at Hofstra/Northwell, Manhasset, New York; North Shore University Hospital, Manhasset, New York; Northwell Health, Manhasset, New York; North Shore University Hospital, Manhasset, New York

## Abstract

**Background:**

Methicillin-resistant Staphylococcus aureus (MRSA) polymerase chain reaction (PCR) of the nares is effective in the screening and detection of MRSA colonization. Prior studies have shown that MRSA PCR has an overall negative predictive value (NPV) of more than 90% for ruling out MRSA infection of various anatomic sites.

**Methods:**

We conducted a retrospective, single-center cohort study to determine if concomitant collection of an MRSA PCR nares swab among patients receiving IV vancomycin in the emergency department (ED) for suspected or known infection alters vancomycin days of therapy (DOT). We educated ED providers to collect MRSA PCR on all patients receiving a dose of vancomycin, however ordering and collection was left at the discretion of the provider. Patients who received at least one dose of IV vancomycin in the ED were identified through a medication usage report and were retrospectively reviewed through their hospital admissions to compare vancomycin DOT in patients who did and did not have an MRSA PCR collected. PCR collection was required to be done by the ED.

**Results:**

Before education, collection of MRSA PCR by ED providers was 0%. Afterwards, there was an observed sustained MRSA PCR collection rate of approximately 25-30%. Of the 236 patients included, 98 had MRSA PCR collected and 138 did not. Both groups showed similar outcomes. There was no statistically significant difference between median vancomycin DOT in the PCR group vs. non-PCR group (3 vs. 2 days, p=0.2638). There was also no statistically significant difference in vancomycin-associated nephrotoxicity (5.10% vs. 5.80%, p=0.9731), hospital length of stay (median 7 days for both groups, p=0.5638), and mortality (10.20% vs. 11.59%, p=0.7368).

**Conclusion:**

Despite the use of MRSA PCR in admitted patients undergoing treatment for suspected infection, there was no significant difference seen in vancomycin DOT or associated secondary outcomes. The utility of MRSA PCR at our institution was likely undermined by shorter vancomycin DOT at baseline, leading to difficulty detecting its impact on vancomycin de-escalation. Given the test’s high NPV, it is likely being underutilized and should be studied further as a tool in the de-escalation of vancomycin.

**Disclosures:**

**All Authors**: No reported disclosures

